# Supplementary material for: Dynalign II: common secondary structure prediction for RNA homologs with domain insertions
Source: Nucleic Acids Res. 2014 Nov 21;42(22):13939–48. doi: 10.1093/nar/gku1172 (PMC4267632; doi:10.1093/nar/gku1172)
Supplement: SUPPLEMENTARY DATA [file supp_gku1172_nar-02021-z-2014-File012.zip › manual/Text/bifold.html]

RNAstructure Command Line Help: bifold


|  |  |  |
| --- | --- | --- |
|  | RNAstructure Command Line Help  bifold and bifold-smp | - Contents - Index |
| bifold is used to predict lowest free energy structures containing two strands. The structures can contain intramolecular pairs. A heuristic is used to predict suboptimal structures. Note that output is written to a CT file where the sequences are concatenated, with an intermolecular linker between them ("III"). bifold-smp is a parallel processing version for use on multi-core computers, built using OpenMP. USAGE: bifold <seq file 1> <seq file 2> <ct file> [options] OR: bifold-smp <seq file 1> <seq file 2> <ct file> [options] Required parameters: <seq file 1> \| The name of a sequence file containing input data for the first sequence. Note that lowercase nucleotides are forced single-stranded in structure prediction.; <seq file 2> \| The name of a sequence file containing input data for the second sequence. Note that lowercase nucleotides are forced single-stranded in structure prediction.; <ct file> \| The name of a CT file to which output will be written. Options which don't require added values: -d, -D, --DNA \| Specify that the sequence is DNA, and DNA parameters are to be used. Note that the backbone type must be the same for both sequences. Default is to use RNA parameters.; -h, -H, --help \| Display the usage details message.; -i, -I, --intramolecular \| Forbid intramolecular pairs (pairs within the same strand). Default is to allow intramolecular pairs. Options which require added values: -l, -L, --loop \| Specify a maximum internal/bulge loop size. Default is 30 unpaired nucleotides.; -m, -M, --maximum \| Specify a maximum number of structures. Note that suboptimal structures are generated until either the maximum number of structures are reached or the maximum percent difference is reached (below). Default is 20 structures.; -p, -P, --percent \| Specify a maximum percent energy difference in free energy of suboptimal structures above the lowest free energy change. Note that suboptimal structures are generated until either the maximum percent free energy difference is reached or until the maximum number of structures is reached (above). Default is 10 percent (specified as 10, not 0.1).; -s, -S, --save \| Specify the name of a save file, needed for dot plots and refolding. Default is not to generate a save file.; -t, -T, --temperature \| Specify the temperature at which calculation takes place in Kelvin. Default is 310.15 K, which is 37 degrees C.; -w, -W, --window \| Specify a window size. Default is 0 nucleotides. Notes for smp: bifold-smp, by default, will use all available compute cores for processing. The number of cores used can be controlled by setting the OMP\_NUM\_THREADS environment variable. References: 1. Reuter, J.S. and Mathews, D.H. "RNAstructure: software for RNA secondary structure prediction and analysis." *BMC Bioinformatics*, 11:129. (2010). 2. Mathews, D.H., Burkard, M.E., Freier, S.M., Wyatt, J.R. and Turner, D.H. "Predicting oligonucleotide affinity to nucleic acid targets." *RNA*, 5:1458-1469. (1999). | | |
| Visit The Mathews Lab RNAstructure Page for updates and latest information. | | |
